# Effectiveness of the 13-valent pneumococcal conjugate vaccine in preventing invasive pneumococcal disease in children aged 7-59 months. A matched case-control study

**DOI:** 10.1371/journal.pone.0183191

**Published:** 2017-08-14

**Authors:** Ángela Domínguez, Pilar Ciruela, Sergi Hernández, Juan José García-García, Núria Soldevila, Conchita Izquierdo, Fernando Moraga-Llop, Alvaro Díaz, Mariona F. de Sevilla, Sebastià González-Peris, Magda Campins, Sonia Uriona, Johanna Martínez-Osorio, Anna Solé-Ribalta, Gemma Codina, Cristina Esteva, Ana María Planes, Carmen Muñoz-Almagro, Luis Salleras

**Affiliations:** 1 Departament de Medicina, Universitat de Barcelona, Barcelona, Spain; 2 CIBER de Epidemiología y Salud Pública (CIBERESP), Madrid, Spain; 3 Agència de Salut Pública de Catalunya, Generalitat de Catalunya, Barcelona, Spain; 4 Malalties Prevenibles amb vacunes, Institut de Recerca Sant Joan de Déu, Esplugues de Llobregat, Spain; 5 Hospital Sant Joan de Déu Barcelona, Universitat de Barcelona, Barcelona, Spain; 6 Hospital Universitari Vall d’Hebron, Barcelona, Spain; 7 Hospital de Nens, Barcelona, Spain; 8 Grup de Recerca en Epidemiologia i Salut Pública, Vall d’Hebron Institut de Recerca, Barcelona, Spain; 9 Departament de Medicina, Universitat Internacional de Catalunya, Barcelona, Spain; Universidade de Lisboa Faculdade de Medicina, PORTUGAL

## Abstract

**Background:**

The 13-valent pneumococcal conjugate vaccine (PCV13) was licensed based on the results of immunogenicity studies and correlates of protection derived from randomized clinical trials of the 7-valent conjugate pneumococcal vaccine. We assessed the vaccination effectiveness (VE) of the PCV13 in preventing invasive pneumococcal disease (IPD) in children aged 7–59 months in a population with suboptimal vaccination coverage of 55%.

**Methods:**

The study was carried out in children with IPD admitted to three hospitals in Barcelona (Spain) and controls matched by hospital, age, sex, date of hospitalization and underlying disease. Information on the vaccination status was obtained from written medical records. Conditional logistic regression was made to estimate the adjusted VE and 95% confidence intervals (CI).

**Results:**

169 cases and 645 controls were included. The overall VE of ≥1 doses of PCV13 in preventing IPD due to vaccine serotypes was 75.8% (95% CI, 54.1–87.2) and 90% (95% CI, 63.9–97.2) when ≥2 doses before 12 months, two doses on or after 12 months or one dose on or after 24 months, were administered. The VE of ≥1 doses was 89% (95% CI, 42.7–97.9) against serotype 1 and 86.0% (95% CI, 51.2–99.7) against serotype 19A. Serotype 3 showed a non-statistically significant effectiveness (25.9%; 95% CI, -65.3 to 66.8).

**Conclusions:**

The effectiveness of ≥1 doses of PCV13 in preventing IPD caused by all PCV13 serotypes in children aged 7–59 months was good and, except for serotype 3, the effectiveness of ≥1 doses against the most frequent PCV13 serotypes causing IPD was high when considered individually.

## Introduction

*Streptococcus pneumoniae* continues to be a leading cause of morbidity and mortality in persons of all ages and the leading cause of bacterial childhood pneumonia and death in children worldwide. Pneumococcal infections include invasive pneumococcal diseases (IPD) such as meningitis, bacteremia and bacteremic pneumonia. In 2015, the estimated number of deaths due to pneumonia worldwide in children aged <5 years was 921,000, and the mortality rate was 6.59 per 1,000 live births [1].

There are currently 97 recognized *S*. *pneumoniae* serotypes [2], but their distribution varies with age and geographical region. The continued incidence and severity of pneumococcal disease, and the increasing prevalence of strains of pneumococci with reduced antimicrobial susceptibility underline the need for research into the prevention of pneumococcal infections.

Pneumococcal protein conjugate vaccines have been designed and developed to prevent invasive and non-invasive pneumococcal disease caused by *S*. *pneumoniae*. The first licensed vaccine, the 7-valent pneumococcal conjugate vaccine (PCV7) contained polysaccharides of seven pneumococcal serotypes (4, 6B, 9V, 14, 18C, 19F and 23F), which were the most frequent IPD-causing serotypes at the end of the last century in the United States. In 2009–2010, two other conjugate vaccines were licensed to respond to emerging serotypes: 10-valent pneumococcal conjugate vaccine (PCV10) (containing serotypes 1, 5 and 7F in addition to the PCV7 serotypes) and PCV13 (containing serotypes 3, 6A and 19A in addition to the PCV10 serotypes) [3].

The PCV13 vaccine, unlike the PCV7 vaccine, was licensed based on the results of immunogenicity studies for the added serotypes and the correlates of protection derived from randomized clinical trials of the PCV7 vaccine [3, 4]. Therefore, continuing observational epidemiological studies (case-control and indirect cohort studies) are required to assess the protective value of the vaccine under normal, non-experimental administration.

The WHO recommends three primary doses or, alternatively, two primary doses plus a booster after considering locally-relevant factors including the epidemiology of pneumococcal disease, the likely coverage and the vaccine schedule of the vaccine doses [5].

Although the Vaccination Advisory Committee of the Spanish Association of Pediatrics has recommended the routine administration of conjugate pneumococcal vaccines (PCV7 since 2003–2010 and, currently, PCV13) these vaccines were not financed by the Catalan Public Health System until July 2016, except in children with selected risk factors, and were only available in the private sector. In Spain, the vaccine was not introduced into the recommended calendar until January 2017, and there are no estimates of vaccination coverage. In the Madrid region, PCV13 was introduced into the vaccination calendar in June 2010 but was later excluded from 2012 to 2015 for economic reasons. During the periods when the vaccine was included in the calendar, the coverage in children aged <2 years was 95% and during the years it was not included, coverage ranged from 67% to 82% [6]. In Navarre, as in Catalonia, the vaccine was not included in the immunization calendar and the coverage was 76% in 2011–2014 in children aged <5 years [7]. In Catalonia, the coverage of PCV13 achieved in children aged <5 years in 2012–2013 was estimated as 55% [8] and the coverage of PCV10 in children aged <2 years was estimated as <4% [9].

Vaccination effectiveness (VE) against PCV13 serotypes requires field investigation because changes in the distribution of serotypes can influence the overall VE. In Catalonia, the overall incidence of IPD and the incidence in children aged <5 years decreased in 2010–2013 in relation to 2006–2009 but the distribution of most frequent serotypes changed [10]. Serotype 3, which had been very infrequent previously [11], has been the most frequent serotype in recent years [10,12] and several failures against IPD caused by this serotype in PCV13-vaccinated children has been observed [8].

The objective of the study was to evaluate the VE of PCV13 against IPD caused by the different serotypes included in the vaccine under usual condition of administration in children aged 7–59 months.

## Methods

### Data confidentiality and ethical aspects

No diagnostic tests were made or samples taken from any participant in addition to those required by routine care. The study complies with the principles of the Declaration of Helsinki and the legal structure in respect to international human rights and biomedicine and protection of personal data laws.

The Ethics Committee of Hospital Sant Joan de Déu approved the study. Informed consent signed by parents or legal guardians was given for all participants (cases and controls). All data were treated as confidential and records were accessed anonymously.

### Study design

A matched case-control study was carried out in patients with IPD admitted to three pediatric hospitals in Barcelona, Spain (Hospital de Nens, Hospital Sant Joan de Déu and Hospital Vall d’Hebrón). The estimated reference population aged <5 years of the three hospitals was 116,279, representing 30.4% of the whole population of this age group in Catalonia (382,507 children) [13]. Cases and controls were recruited between January 2012 and June 2016.

### Selection of cases

Patients aged 7–59 months hospitalized for IPD were studied. IPD was defined as isolation by culture of *S*. *pneumoniae* or DNA detection of the Lyt A gene and an additional capsular gene of *S*. *pneumoniae* by real-time polymerase chain reaction (PCR) in any normally-sterile site according to a previously reported method [14]. Strains isolated by culture were serotyped using the Quellung reaction or dot blot by the National Centre for Microbiology, Majadahonda, Madrid. Culture-negative and PCR-positive samples with a cycle threshold (Ct) >30 cycles were serotyped using a previously-described, real-time multiplex PCR technique [15]. PCR-positive samples with a Ct ≤30 cycles were serotyped using sequential multiplex PCR combined with fragment analysis and automated fluorescent capillary electrophoresis [16]. Children with IPD in whom serotyping of *S*. *pneumoniae* was not possible because the sample was not available were excluded.

### Selection of controls

Four controls were selected for each case from patients attending the same hospitals for causes other than IPD. Controls were matched by age (±6 months in patients aged <12 months and ±12 months in patients aged 12–59 months), sex, date of hospitalization of the case (±3 months) and underlying medical conditions [17], when present. Study investigators were blinded to the vaccination status of all controls during selection.

### Vaccination status of cases and controls

A case or control was considered vaccinated if this was recorded in the medical record, the vaccination card or the child’s health card. Cases and controls whose vaccination status could not be determined were excluded. Any dose of PCV13 given after six weeks of age, at least four weeks after the previous dose, and at least 15 days before the hospital admission date (cases) or before the hospitalization date of the matched case (controls) was considered valid.

To assess the VE against all PCV13 serotypes, the vaccination status included only doses of PCV13 and any child (case or control) who received PCV7, PCV10 or a mixture of either with PCV13 was excluded from the analysis. To assess the VE against PCV13-non-PCV7 serotypes, only doses of PCV13 were considered and any child who received only PCV7 or PCV10 was excluded from the analysis.

### Sociodemographic, clinical and epidemiological variables

The demographic and clinical variables recorded for each case were: age, sex, date of hospitalization, clinical form, underlying medical conditions, antibiotic therapy, history of respiratory infection in the 30 days before symptom onset and recurrent acute otitis media. Other epidemiological variables recorded were: birth weight, day care or school attendance, house size, number of cohabitants, siblings, parental smoking, parental education and influenza vaccination in the current season. The same variables were recorded for controls except for those relating to IPD. All variables were collected using a single questionnaire for cases and controls and there was an instruction manual to facilitate compliance.

### Sample size

The sample size required was calculated using Schlesselman’s criteria [18]. Assuming a prevalence of a history of vaccination in controls of 47% [19], a VE of 80%, a bilateral α error of 0.05 (two-tailed), and a β error of 0.2, and supposing that 25% of cases would be caused by vaccine serotypes (at present the figure is higher but vaccine serotypes are expected to decrease following the introduction of PCV13), that four controls would be sought per case, and that children aged 7–23 months and those aged 24–59 months would be analyzed separately, the minimum number of cases required was estimated at 160 and the number of controls as 642.

### Statistical analysis

Differences in demographic, clinical and epidemiological variables between cases and controls were analyzed using the McNemar test for categorical variables and the paired t-test for continuous variables. A two-tailed distribution was assumed for all p-values.

To estimate the adjusted VE a multivariate analysis was performed using conditional logistic regression. We introduced all demographic, clinical and epidemiological variables in the model and obtained a final model using backward stepwise selection with a cut-off point of p<0.2. Interactions between vaccination and other variables were assessed using the likelihood ratio test in the conditional regression analysis. Independent variables were checked for collinearity using the variance inflation factor [20].

VEwascalculatedusingtheformulaVE=(1−OR)×100.

Analyses were performed for PCV13 serotypes, PCV13-non-PCV 7 serotypes, and individual serotypes according to the number of doses, schedule and age group. The analysis was performed using the SPSS v24 statistical package and R v3.3.0 statistical software.

## Results

**Fig 1:**
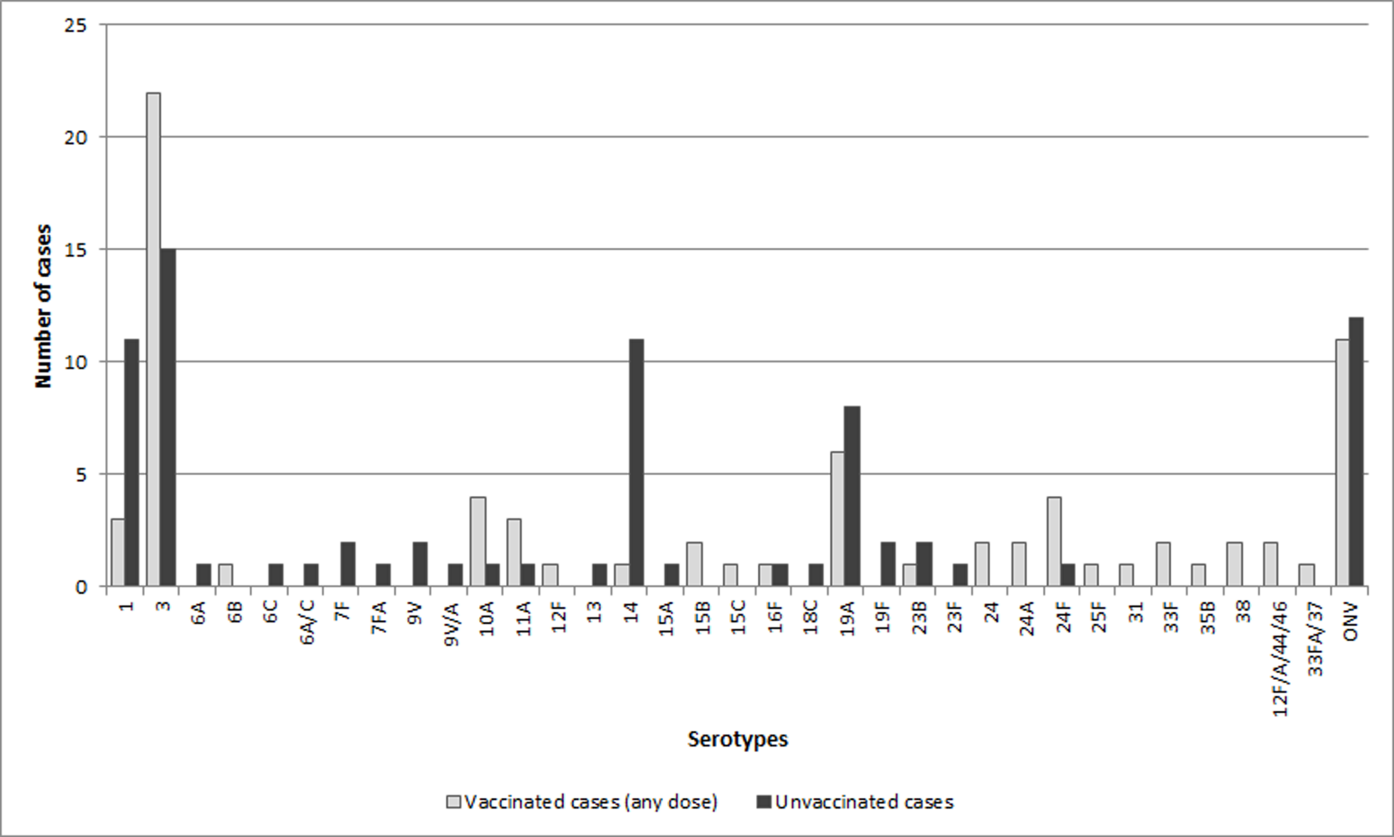
Distribution of serotypes of cases included in the study according to the receipt of any dose of PCV13 vaccine^a^. ONV = Other non-PCV13 serotypes ^a^Any child who received only PCV7 or PCV10 was excluded

During the study period, 180 cases of IPD were detected in patients aged 7–59 months, of whom 169 were included. The remaining cases were excluded because no controls meeting the study criteria were found (one case), or the sample for serotyping (seven cases) or the clinical record (three cases) were not available. A total of 239 samples from the 169 patients were studied. Of these, 31 samples were negative and 208 were positive for PCR and/or culture. Of the 144 blood samples analyzed, 43 were positive for culture alone, 49 for PCR alone and 23 for culture and PCR; of the 74 pleural fluid samples, 4 were positive for culture alone, 56 for PCR alone and 13 for culture and PCR; of the 12 cerebrospinal fluid samples, 3 were positive for culture alone, 4 for PCR alone and 4 for culture and PCR; all 4 synovial fluid samples, 1 epidural abscess sample and 1 otogenic abscess sample analyzed were positive for culture alone; 2 mastoid samples and 1 nasopharyngeal aspirate sample were positive for PCR alone. Cases with nasopharyngeal aspirate and otogenic abscess samples also had positive blood samples. Of the 169 cases, 83 (49.1%) were diagnosed by PCR alone, 43 (25.4%) by culture alone and 43 (25.4%) by culture and PCR. The most frequent serotypes in the cases included were serotype 3 (24.3%), serotype 1 (11.2%), serotype 19 A (9.5%) and serotype 14 (7.1%). The distribution of serotypes according to the receipt of any dose of PCV13 vaccine is shown in Fig 1. Serotype 3 was more frequent in the 24–59 months age group than in the 7–23 months age group (75.6% vs. 24.4%, p = 0.01). The clinical manifestations of the cases were: complicated pneumonia including empyema, pleural effusion or necrotizing pneumonia (58.6% in all cases, 45.1% in children aged 7–23 months and 68.4% in children aged 24–59 months), uncomplicated pneumonia (15.4% in all cases, 14.1% in children aged 7–23 months and 16.3% in children aged 24–59 months), occult bacteremia (10.6% in all cases, 16.9% in children aged 7–23 months and 6.1% in children aged 24–59 months), and meningitis (6.5% in all cases, 9.9% in children aged 7–23 months and 4.1% in children aged 24–59 months). Other forms included mastoiditis (3.0%), bone and joint infection (3.0%), septic shock (2.3%) and cellulitis (0.6%).

We included 645 matched controls attending the same hospitals due to causes other than IPD. The demographic, epidemiologic and clinical characteristics of cases and controls are shown in Table 1. The proportion of cases and controls who had received ≥1 doses of PCV13 were 44.4% and 63.6%, respectively. The underlying medical conditions of cases and controls were: immunosuppressive therapy (4 cases and 9 controls), chronic renal failure (1 case and 3 controls), chronic lung disease (1 case and 3 controls) and chronic heart disease (2 cases and 2 controls). No interaction was found between vaccination and the other variables. Crude and adjusted estimations of VE for all PCV13 serotypes and all PCV13 serotypes, excluding serotype 3, according to age, number of doses and vaccination schedule is shown in Table 2. Table 3 summarizes the estimates of crude and adjusted VE for PCV13-non-PCV7 serotypes, including and excluding serotype 3, according to age, number of doses and vaccination schedule. The VE against individual serotypes 1, 3, 14 and 19A is shown in Table 4 (this could not be assessed for other serotypes due to the low number of cases).

**Table 1:** Characteristics of cases and controls.

Characteristic	Cases	Controls	P value
	(N = 169)	(N = 645)	
**Age in months,** median (range)	27 (7–59)	29 (7–71)	0.04
**Age group**			0.20
7–23 months	71 (42.0%)	252 (39.1%)	
24–59 months	98 (58.0%)	393 (60.9%)	
**Sex**			1.00
Female	64 (37.9%)	248 (38.4%)	
Male	105 (62.1%)	397 (61.6%)	
**Underlying medical condition**			1.00
Yes	8 (4.7%)	17 (2.6%)	
No	161 (95.3%)	628 (97.4%)	
**Birth weight,** median (range)	3.2 (0.8–5.6)	3.2 (0.62–5.0)	0.08
**Breastfeeding**			0.21
Yes	140 (82.8%)	508 (78.8%)	
No	29 (17.2%)	137 (21.2%)	
**Day care or school attendance**			0.01
Yes	120 (71.0%)	411 (63.7%)	
No	49 (29.0%)	234 (36.3%)	
**Antibiotic treatment in previous month**			0.71
Yes	26 (15.4%)	104 (16.1%)	
No	143 (84.6%)	541 (83.9%)	
**Respiratory infection in previous month**			0.51
Yes	96 (56.8%)	354 (54.9%)	
No	73 (43.2%)	291 (45.1%)	
**Recurrent acute otitis media**			0.26
Yes	24 (14.2%)	116 (18.0%)	
No	145 (85.8%)	529 (82.0%)	
**House size (m2),** median (range)	80 (40–400)	85 (40–500)	0.01
**Cohabitants,** median (range)	4 (2–10)	4 (1–11)	0.62
**Siblings**			0.41
Yes	95 (56.2%)	386 (59.8%)	
No	74 (43.8%)	259 (40.2%)	
**Exposure to tobacco in the home**			0.34
Yes	55 (32.5%)	233 (36.1%)	
No	114 (67.5%)	412 (63.9%)	
**Head of the family working**			0.33
Yes	151 (89.3%)	593 (91.9%)	
No	18 (10.7%)	52 (8.1%)	
**Influenza vaccine in the current season**			0.20
Yes	3 (1.8%)	24 (3.7%)	
No	166 (98.2%)	621 (96.3%)	
**Educational level of parents**^**a**^			0.27
No or primary	16 (10.1%)	50 (8.0%)	
Secondary or higher	143 (89.9%)	575 (92.0%)	
**≥1 dose of PCV13 (7–59 months)**	75 (44.4%)	410 (63.6%)	<0.01
7–23 months	38 (53.5%)	180 (71.4%)	<0.01
24–59 months	37 (37.8%)	230 (58.5%)	<0.01

PCV13 = 13-valent pneumococcal conjugate vaccine

^a^This information was not available for 10 cases and 20 controls

**Table 2:** Crude and adjusted effectiveness of the 13-valent pneumococcal conjugate vaccination against IPD caused by PCV13 serotypes including and excluding serotype 3 in the prevention of invasive pneumococcal disease in children aged 7–59 months according to vaccination schedule, number of doses received and age.

Serotypes coverage, vaccination schedule and age groups		Cases Vaccinated/N (%)	Controls Vaccinated/N (%)	Crude VE (95% CI)	p value	Adjusted VE (95% CI)	p value
**All PCV13 serotypes**^**a**^							
	≥1 dose	29/85 (34.1%)	189/298 (63.4%)	69.4% (48.3–81.9)	<0.001	75.8% (54.1–87.2)	<0.001
	7–23 months	10/31 (32.3%)	82/121 (67.8%)	75.1% (43.9–88.9)	0.001	80.7% (45.3–93.2)	0.002
	24–59 months	19/54 (35.2%)	107/177 (60.5%)	64.4% (29.6–82.0)	0.003	75.3% (45.4–88.9)	0.001
	≥2 doses before 12 months or 2 doses on or after 12 months or 1 dose on or after 24 months	13/69 (18.8%)	67/176 (38.1%)	65.9% (24.8–84.5)	0.01	90.0% (63.9–97.2)	<0.001
	7–23 months	6/27 (22.2%)	51/90 (56.7%)	76.7% (36.6–91.4)	0.004	92.8% (55.4–98.8)	0.005
	24–59 months	7/42 (16.7%)	16/86 (18.6%)	32.5% (-127.7–80.0)	0.53	83.5% (-19.5–97.7)	0.07^b^
	≥2 doses before 12 months and 1 dose after 12 months	15/71 (21.1%)	116/225 (51.6%)	70.3% (42.2–84.8)	<0.001	78.9% (52.8–90.5)	<0.001
	7–23 months	3/24 (12.5%)	31/70 (44.3%)	79.4% (22.1–94.5)	0.02	91.2% (16.2–99.9)	0.03
	24–59 months	12/47 (25.5%)	85/155 (54.8%)	65.9% (26.0–84.3)	0.01	73.4% (36.4–88.9)	0.003
**All PCV13 serotypes, excluding serotype 3**^**a**^							
	≥1 dose	9/50 (18.0%)	112/175 (64.0%)	87.7% (72.3–94.5)	<0.001	95.8% (84.0–98.9)	<0.001
	7–23 months	6/22 (2.7%)	63/86 (73.3%)	83.5% (55.5–93.9)	<0.001	95.5% (54.5–99.5)	0.01
	24–59 months	3/28 (10.7%)	49/89 (55.1%)	92.8% (67.4–98.4)	<0.001	97.1% (79.8–99.6)	<0.001
	≥2 doses before 12 months or 2 doses on or after 12 months or 1 dose on or after 24 months	5/46 (10.9%)	46/109 (42.2%)	83.9% (48.8–95.0)	0.002	97.9% (75.3–99.8)	0.002
	7–23 months	4/20 (20.0%)	38/61 (62.3%)	80.7% (35.2–94.2)	0.008	92.7% (35.3–99.2)	0.02
	24–59 months	1/26 (3.8%)	8/48 (16.7%)	-	-	-	-
	≥2 doses before 12 months and 1 dose after 12 months	3/44 (6.8%)	63/126 (50.0%)	91.8% (71.9–97.6)	<0.001	97.5% (83.9–99.6)	<0.001
	7–23 months	1/17 (5.9%)	25/48 (52.1%)	93.4% (45.4–99.2)	0.01	96.9% (58.7–99.8)	0.01
	24–59 months	2/27 (7.4%)	38/78 (48.7%)	90.7% (57.6–98.0)	0.002	95.1% (68.0–99.3)	0.002

CI = Confidence interval; IPD = Invasive pneumococcal disease; PCV13 = 13-valent pneumococcal conjugate vaccine; VE = vaccine effectiveness.

^a^Any child who received PCV7, PCV10 or a mix of either with PCV13 was excluded. ^b^Statistical power: 88%

**Table 3:** Crude and adjusted effectiveness of the 13-valent pneumococcal conjugate vaccination against IPD caused by PCV13-non-PCV7 serotypes including and excluding serotype 3 in children aged 7–59 months according to vaccination schedule, number of doses received and age.

Serotypes coverage, vaccination schedule and age groups		Cases Vaccinated/N (%)	Controls Vaccinated/N (%)	Crude VE (95% CI)	p value	Adjusted VE (95% CI)	p value
**PCV13-non-PCV7 serotypes**^**a**^							
	≥1 dose	31/70 (44.3%)	165/253 (65.2%)	56.9% (25.5–75.1)	0.003	64.2% (31.9–81.2)	0.002
	7–23 months	9/22 (40.9%)	55/86 (63.9%)	59.0% (-4.3–83.9)	0.06	54.3% (-37.7–84.8)	0.16^b^
	24–59 months	22/48 (45.8%)	110/167 (65.9%)	55.8% (13.0–77.6)	0.02	66.9% (28.6–84.7)	0.005
	≥2 doses before 12 months or 2 doses on or after 12 months or 1 dose on or after 24 months	13/52 (25.0%)	50/138 (36.2%)	46.8% (-21.9–76.7)	0.14	76.1% (26.3–92.2)	0.01
	7–23 months	5/18 (27.8%)	34/65 (52.3%)	65.6% (-8.7–89.1)	0.07	89.8% (24.2–98.6)	0.03
	24–59 months	8/34 (23.5%)	16/73 (21.9%)	13.3% (-175.2–72.7)	0.81	70.4% (-102.3–95.7)	0.21^c^
	≥2 doses before 12 months and 1 dose after 12 months	14/53 (26.4%)	93/181 (51.4%)	59.4% (16.6–80.3)	0.01	70.0% (30.1–87.1)	0.01
	7–23 months	3/16 (18.8%)	21/52 (40.4%)	62.3% (-53.7–90.7)	0.17	58.7% (-126.9–92.5)	0.31^d^
	24–59 months	11/37 (29.7%)	72/129 (55.8%)	58.3% (3.5–82.0)	0.04	71.4% (25.6–89.0)	0.01
**PCV13-non-PCV7 serotypes, excluding serotype 3**^**a**^							
	≥1 dose	9/33 (27.3%)	74/113 (65.5%)	81.3% (53.8–92.5)	<0.001	95.7% (75.7–99.3)	<0.001
	7–23 months	5/13 (38.5%)	36/51 (70.6%)	70.8% (3.2–91.2)	0.04	90.1% (34.8–98.5)	0.02
	24–59 months	4/20 (20.0%)	38/62 (61.3%)	89.7% (51.9–97.8)	0.004	95.3% (64.0–99.4)	0.003
	≥2 doses before 12 months or 2 doses on or after 12 months or 1 dose on or after 24 months	4/28 (14.3%)	28/67 (41.8%)	76.5% (6.6–94.1)	0.04	95.5% (38.4–99.7)	0.02
	7–23 months	3/11 (27.3%)	21/36 (58.3%)	66.9% (-49.5–92.7)	0.15	95.0% (-0.1–99.8)	0.05^e^
	24–59 months	1/17 (5.9%)	7/31 (22.6%)	-	-	-	-
	≥2 doses before 12 months and 1 dose after 12 months	2/26 (7.7%)	39/78 (50.0%)	90.5% (56.6–97.9)	0.002	97.9% (69.9–99.9)	0.005
	7–23 months	1/9 (11.1%)	15/30 (50.0%)	87.0% (-19.6–98.6)	0.07	93.1% (2.3–99.5)	0.04
	24–59 months	1/17 (5.9%)	24/48 (50.0%)	92.5% (38.3–99.1)	0.02	98.5% (58.4–99.9)	0.01

CI = Confidence interval; IPD = Invasive pneumococcal disease; PCV13 = 13-valent pneumococcal conjugate vaccine; PCV7 = 7-valent pneumococcal conjugate vaccine; VE = vaccine effectiveness.

^a^Any child who received only PCV7 or PCV10 was excluded.

Statistical power: ^b^ 71%

Statistical power: ^c^ 69%

Statistical power: ^d^ 52%

Statistical power: ^e^ 90%

**Table 4:** Crude and adjusted effectiveness of the 13-valent pneumococcal conjugate vaccination against IPD in children aged 7–59 months by specific serotype, vaccination schedule, and number of doses received.

Serotypes coverage, vaccination schedule and age groups	Cases Vaccinated/N (%)	Controls Vaccinated/N (%)	Crude VE (95% CI)	p value	Adjusted VE (95% CI)	p value
**Serotype 1**^**a**^						
≥1 dose	3/14 (21.4%)	30/45 (66.7%)	88.4% (42.6–97.7)	0.01	89.0% (42.7–97.9)	0.01
≥2 doses before 12 months or 2 doses on or after 12 months or 1 dose on or after 24 months	0/11 (0.0%)	6/21 (28.6%)	-	-	-	-
≥2 doses before 12 months and 1 dose after 12 months	1/12 (8.3%)	19/34 (55.9%)	91.9% (31.1–99.0)	0.02	91.0% (12.2–99.1)	0.04
**Serotype 3**^**a**^						
≥1 dose	22/37 (59.5%)	91/140 (65.0%)	19.6% (-68.8–61.7)	0.56	25.9% (-65.3–66.8)	0.46^c^
≥2 doses before 12 months or 2 doses on or after 12 months or 1 dose on or after 24 months	9/24 (37.5%)	22/71 (31.0%)	4.4% (-179.6–67.3)	0.93	63.3% (-56.2–91.4)	0.17^d^
≥2 doses before 12 months and 1 dose after 12 months	12/27 (44.4%)	54/103 (52.4%)	7.5% (-127.9–62.4)	0.87	12.8% (-127.9–66.6)	0.78^e^
**Serotype 14**^b^						
≥1 dose	1/12 (8.3%)	34/46 (73.9%)	95.7% (65.2–99.5)	0.003	96.9% (70.4–99.7)	0.003
≥2 doses before 12 months or 2 doses on or after 12 months or 1 dose on or after 24 months	0/11 (0.0%)	14/26 (53.8%)	-	-	-	-
≥2 doses before 12 months and 1 dose after 12 months	1/12 (8.3%)	20/32 (62.5%)	92.0% (31.1–99.1)	0.02	94.2% (41.8–99.4)	0.01
**Serotype 19A**^**a**^						
≥1 dose	6/14 (42.9%)	35/50 (70.0%)	67.7% (-7.7–90.3)	0.07	86.0% (51.2–99.7)	0.01
≥2 doses before 12 months or 2 doses on or after 12 months or 1 dose on or after 24 months	4/12 (33.3%)	20/35 (57.1%)	68.9% (-38.1–93.0)	0.12	85.6% (6.7–99.8)	0.04
≥2 doses before 12 months and 1 dose after 12 months	1/9 (11.1%)	14/29 (48.3%)	83.3% (-62.3–98.3)	0.12	84.1% (-97.1–98.7)	0.15^f^

CI = Confidence interval; IPD = Invasive pneumococcal disease; VE = vaccine effectiveness.

^a^Any child who received only PCV7 or PCV10 was excluded.

^b^Any child who received PCV7, PCV10 or a mix of either with PCV13 was excluded.

Statistical power: ^c^ 23%

Statistical power: ^d^ 67%

Statistical power: ^e^ 7%

Statistical power: ^f^ 64%

## Discussion

This matched case-control study carried out in children aged 7–59 months admitted to three Catalonia pediatric hospitals has provided three main results. First, the overall VE of ≥1 doses of PCV13 in preventing IPD caused by vaccine serotypes is good (75.8%; 95% CI, 54.1–87.2) and increases when ≥2 doses before 12 months, two doses on or after 12 months or one dose on or after 24 months, are administered (90%; 95% CI, 63.9–97.2). In the 24–59 months age group, the latter schedule showed a non-significant effectiveness against IPD (83.5%; 95% CI, -19.5 to 97.7), probably because serotype 3 was more frequent in this age group. Secondly, the VE of ≥1 doses against two previously-emergent serotypes in our geographical area was high: 89% (95% CI, 42.7–97.9) for serotype 1 and 86.0% (95% CI, 51.2–99.7) for serotype 19A, but the VE of ≥1 doses against serotype 3 showed a non-significant effectiveness (25.9%; 95% CI, -65.3 to 66.8). Thirdly, the VE of ≥1 doses against serotype 14 was the highest found (96.9%; 95% CI, 70.4–99.7), which is important because this serotype is a very frequent cause of IPD in Catalonia [12].

Our results are similar to those obtained in observational studies carried out in other locations [7,21–27]. In case-control studies [7,21–26], the point estimate of VE against PCV13 serotypes ranges between the 77% (95% CI, 38–91) found by Miller et al. [21] in the United Kingdom in children aged 2.5–24 months who received one dose on or after 12 months or at least one dose before 12 months and one dose on or after 12 months and the 91% (95% CI, 61–99) found by Van Der Linden et al.[23] in children aged <2 years who had received the complete immunization schedule in Germany. In the study by Cohen et al. [26] in South Africa, the distribution of IPD-causing serotypes was very different from that found in Catalonia, because serotype 3 was not frequent and HIV infection is very frequent; however in non-HIV infected children, the VE for PCV13-non-PCV7 serotypes was 92% (95% CI, 40–99), very close to the 95.5% (95% CI, 38.4–99.7) obtained in our study when serotype 3 was excluded from the analysis.

The VE of serotype 19A, the third most frequent serotype in our study, was 86.0% (95% CI, 51.2–99.7) for children receiving ≥1 doses of PCV13 and the results were similar for children receiving ≥2 doses before 12 months, two doses on or after 12 months or one dose on or after 24 months (85.6%; 95% CI, 6.7–99.8). The fact that the VE confidence intervals in children receiving ≥2 doses before 12 months and one dose after 12 months included zero might be explained by the low number of cases. In the study by Miller et al. [21] the VE against serotype 19 was 70% (95% CI, 10–90) and in the study by Van Der Linden et al. [23] it was 88% (95% CI, 25–99). In the study by Deceuninck et al. [22] the VE observed against serotype 19A was not statistically-significant (68%; 95% CI, -13 to 91). In the indirect cohort study by Andrews et al. [27] in England, Wales and Northern Ireland, the VE was 67% (95% CI, 33–84) for this serotype.

Studies carried out in Western European countries from 2010 to 2014 [28], in Germany during 1992 to 2014 [29], in the United States during 2004 to 2013 [30], in Portugal from 2012 to 2014 [31] and in England and Wales from 2008 to 2014 [32] have assessed the impact of PCV13 vaccination on the population and their results confirm those of case-control and indirect cohort studies [28–32]. In most countries that introduced the PCV13, large reductions in the incidence of IPD have been observed for all vaccine serotypes and for PCV13-non-PCV7 serotypes [28–32]. The reductions were even higher in the cases of serotype 19A in both children and adults aged >65 years, confirming herd immunity for this serotype [28–33]. Previous immunogenicity studies had forecast a high level of effectiveness against this serotype [27,34].

An important finding of our study is that the VE against all PCV13 serotypes and against PCV13-non-PCV7 serotypes increased for all vaccination schedules and age groups when serotype 3 cases were excluded from the analysis. Before the PCV13 was licensed, some authors had suggested that this serotype was less immunogenic than the other PCV13 serotypes [35]. In a Canadian study carried out after complete PCV13 vaccination in children, the level of opsonophagocitic antibodies was lower against serotype 3 than against other serotypes, suggesting the future level of protection would also be also low [36]. In a study carried out in Spain and Poland, the immune response against serotype 3 was also clearly lower than that obtained against other serotypes, particularly serotype 19A [37]. In a double-blinded trial comparing the efficacy of the PCV7 with that of PCV13 in preventing *S*. *pneumoniae* nasopharyngeal colonization, different levels of colonization were found in vaccinated and unvaccinated children for the serotypes studied, except for serotype 3 [38].

No VE of PCV13 was found for serotype 3 in two case-control studies [21,23] and one indirect cohort study [27]. Significant protection (79.5%; 95% CI, 30.3–93.1) by PCV13 against serotype 3 was observed only in the matched case-control study by Moore et al. [24], but this was lower than that found for serotypes 7F (96.5%; 95% CI, 82.7–100) and 19A (85.6%; 95% CI, 70.6–93.5). As in the present study, Andrews et al. [27] found that the VE of all serotypes was statistically significant except for serotype 3 (VE 26%, 95% CI, -69 to 68). According to these authors, the immune correlate of protection of this serotype (2.83 μg/mL, which was rarely attained with vaccination), was higher than the threshold of 0.35 μg/mL established for the PCV7 serotypes.

In most western European countries, IPD caused by serotype 3 has been stable or has increased after the introduction of the PCV13 [28,39]. In Canada and Japan, a similar trend has been observed [33,40]. Interestingly, the impact study by Moore et al. [30] found no reduction in the disease incidence caused by serotype 3 in the population, although the same authors had found that PCV13 was effective against serotype 3 in a case-control study [24]. The study by Waight et al. in the United Kingdom found some reduction in the incidence of IPD caused by serotype 3 in all age groups, but in children aged <5 years, the confidence intervals were very wide (68%; 95% CI, 0.6–89) [32].

In the present study, 44.4% (12/27) of children with IPD caused by serotype 3 had received ≥2 vaccine doses before 12 months and one dose after 12 months. These results are in accordance with those found in Greece [41], where a third of serotype 3 cases who presented pneumonia with pleural effusion were completely vaccinated.

This case-control study, like all observational studies, could be subject to selection and information biases: selection bias was minimized because controls were matched with cases by age, sex, risk medical conditions, hospital and date of case admission, and information bias was minimized because data were obtained in the same way in cases and controls. It seems unlikely that information bias may invalidate our results because information on the vaccination status was obtained from health records completed before study recruitment. In addition, variables that could confound the results were introduced into the conditional regression analysis. However, some residual confounding cannot be ruled out.

A strength of the study was the diagnosis by PCR: nearly half the cases would not have been detected if only cultures had been used and, therefore, the cases included would not have been representative of hospitalized cases of IPD.

A limitation of the study is that the VE against clinical forms other than pneumonia or complicated pneumonia and against serotypes other than serotypes 1, 3, 14 and 19A could not be estimated due to the low number of cases.

In conclusion, we found that the effectiveness of ≥1 doses of PCV13 against IPD caused by PCV13 serotypes in children aged 7–59 months was good (≥75.8%) and that the effectiveness increased for all types of vaccination schedules and age groups when serotype 3 cases were excluded from the analysis. The VE of ≥1 dose for serotypes 1, 14 and 19A, the most frequent in our area after serotype 3, is high, ranging between 86% and 96.9%.
